# Ethics in Palliative Care

**DOI:** 10.4103/0973-1075.58450

**Published:** 2009

**Authors:** Bidhu K Mohanti

**Affiliations:** Department of Radiation Oncology, Dr. BRA Institute Rotary Cancer Hospital, AIIMS, New Delhi - 110 029, India E-mail: drbkmohanti@rediffmail.com

Physicians and nurses encounter difficulties in their practice of palliative Care. They do need a good understanding of ethical principles and precedents.[1,2]

‘A 60-year-old lady with relapsed ovarian cancer, with ascites and profound dyspnea, is in her terminal stage of disease. She has this disease progression one year after her surgery and six months after chemotherapy. Her husband is a retired 65-year-old teacher with chronic heart disease. The son and daughter stay at distant places and can only visit frequently’.

There are a wide range of medical issues and ethical dilemmas that arise in the provision of palliative care for this lady. It is now realized that a good understanding of medical ethics will contribute to the health professional's decision-making and day-to-day practice of medicine for a terminally ill patient.[2]

Medical ethics is primarily a field of applied ethics, the study of moral values and judgments as they apply to medicine. It is intended to provide guidelines and codes for physicians as for their *duty, responsibility* and *conduct* and shares many principles with other health care ethics, such as nursing ethics and bioethics.[3] Historically, it can be traced back to Hippocrates, the ancient Greek physician of 4^th^ century BC. Hippocrates (460-380 BC) and his school of students set themselves apart from other healers of their time by stressing that their professional pursuits were rational and scientific rather than magical or religious.[4] Several medical thinkers have emphasized that a physician should carry *‘a good sense and discretion’*.

From the 18^th^ century onwards, medical practitioners adopted the Hippocratic Oath as the rite of passage. Similarly, the guiding principle of nursing profession is known as Nightingale Pledge.[3] These two, for the medical and nursing profession respectively, are enshrined in part as, *‘I will prescribe regimens for the good of my patients according to my ability and my judgment and never do harm to anyone’*, and *‘I will practice my profession with conscience and dignity’*.

Major evolution and developments in medical ethics were made since 20^th^ century. The betrayal of scientific medicine's ideals during the Second World War by Nazi doctors, in carrying out inhuman medical practices amounting to torture and killing of innocent prisoners, lead to the Nuremberg trial in 1947. The Nuremberg code was issued and this is considered the basis of modern medical ethics.[5] Treatment of human subjects, informed consent, protecting research subjects from harmful medical experiments were incorporated as laws of the society. These laws and policies are provided by Declaration of Geneva (1948, last modified in 2006) and further incorporated in Declaration of Helsinki (1964, last modified in October 2008).[6] It is the responsibility of all medical practitioners to read these documents and imbibe the stated values.

## SIX VALUES IN MEDICAL ETHICS[1]

The foundation of medical ethics is supported by four pillars, namely;

*Autonomy* - patient has the right to choose or refuse the treatment

*Beneficence* - a doctor should act in the best interest of the patient

*Non-maleficence* - first, do no harm

*Justice* - it concerns the distribution of health resources equitably.

Added to the above four, are two more aspects which form the cornerstones of medical practice:

*Dignity* - the patient and the persons treating the patient have the right to dignity

*Truthfulness and honesty* - the concept of informed consent and truth telling

All these together constitute the six values of medical ethics.

## INTEGRATING MEDICAL ETHICS INTO PALLIATIVE CARE

The lady with ovarian cancer, narrated at the beginning (who had earlier been treated by chemotherapy) could be offered second-line of aggressive chemotherapy. Is this the only option? Often the treating oncology team may be reluctant to discuss the *risk/benefit* aspects of second-line and may not consider the option of palliative care in the face of progressive ascites and pleural effusion.

This exemplifies the need for patient's autonomy, beneficence vs. non-maleficence, and truth telling. It is pertinent to understand the clinical decision making process of ‘withholding or withdrawing a treatment’ (in this case the second-line chemotherapy with negligible response). E. Ezekiel of Bioethics department, NIH, USA has expressed concerns about the quality of care that cancer patients get, ‘physicians give patients, who are not responding, different aggressive therapy, “Just to try something.” That is just not bad medicine, it is seriously unethical’.[7]

It is known that timely institution of palliative care alleviates the distressing symptoms in terminal stages of diseases, avoids toxicities of questionable anti-cancer therapy, and improves the quality of remaining life. The treating palliative care team may face conflicts in terms of patient's family carer refusing to stop the toxic anti-cancer therapy. Hence effective communication (and explanation of the disease process) is the key to ethical palliative care.

## PATIENT HAS A RIGHT TO KNOW

The team should be knowledgeable to give proactive care, understand the patient's preferences and forgive conflicts. The process of truth telling in advanced cancer or any other terminal illness can be a difficult task. Whenever a patient is too moribund and not in a suitable mental stage, the family carers are required to give informed consent. The doctor and nurse in the palliative care team have to build the communication with a responsible family carer so that confidentiality and dignity for patient's last stage are maintained.[1,2] Communication is meant to deal with ethical questions regarding two fundamental aspects of Palliative Care: To explain the concept of a good death and to resolve the conflicting needs of patient vis-à-vis family.[8]

The lady with progressive ovarian cancer has fluid in abdomen and chest, and may not get a regular appetite. The husband could feel distressed that she is going to die in hunger. This needs a good explanation to alleviate the conflict regarding forced feeding in a terminal stage of cancer. Effective and compassionate communications are the integral components of ethics are in palliative care.[2,8]

## DEMANDS FOR HEALTH RESOURCES IN PALLIATIVE CARE

Pain is the dominant symptom for many advanced stage cancer patients and so also for other chronic illness like HIV/AIDS.[9] Pain can make a patient fearful, withdrawn, and agitated and unrelieved pain leads to a miserable death leaving the family members remorseful in grief. Pain relief is successfully achieved by the scientific and holistic principles of analgesic ladder in palliative care.[8] Whereas relief of pain is a core ethical duty in medicine, it is observed that pain is the most neglected aspect in medical care.[10] There are several interlinked causes for this neglect. Lack of knowledge and skill in pain assessment, improper medication, unavailability of morphine, unfounded myths about opioid addiction and sedation are some of the complex hurdles. The efforts of the World Health Organization (WHO) and various national bodies/associations of palliative care improved the situation. It is now recognized in most countries that relief from pain is a legal right and availability of morphine is a societal responsibility. For ethical reasons, the correct step would be to view pain as a public health crisis, and take the necessary steps to remove all hurdles.[9,10]

The public fear that drugs such as sedatives and opioids prescribed in the terminal stage of a patient hasten the death process.[8,9] The competent medical practitioners should dispel this myth. It is ethical to prescribe narcotics and sedatives for intractable pain, even when there is the possibility of *terminal sedation*. Such a possibility is called ‘double effect’ and the treating team should explain this aspect to the family members. Often metaphors can be highly effective, *‘we had a patient with lung cancer who suffered from breathing difficulty and pain; he was constantly breathless, could not lie down, and did not sleep for nights together. Morphine calmed him down; he felt relieved, could sleep soundly, and passed away peacefully after two weeks. His wife felt a sense of relief when he could sleep well’*.

End-of-life care is both a medical and an ethical challenge.[2,7–9] Patients and their family can face several uncertainties. In the last part of life, with multiple distressing symptoms, infection, anorexia-cachexia, fatigue, mental confusion etc, deciding the right place of care is the first priority. Whenever possible, a good death is when it comes at the patient's home, surrounded by family members and relatives. Hence, *advance care planning* should be recorded.[8] As far as possible, home care instead of hospital or hospice should be explained by the palliative care team. In India, home care will be less expensive and a more practical approach to offer palliative care at the door-step. There can crop up certain contentious issues like use of antibiotics, supportive drugs, blood transfusion, naso-gastric tube, parenteral nutrition, intensive care etc. Wherever possible, the patient's preferences should be balanced with palliative care principles. Advance directives in the form of recording the patient's or family's consent should be routinely practiced. This will avoid the terminal palliative care patient being subjected to unnecessary tests, hospitalization, intensive monitoring, and resuscitation procedure. In many countries of the world, do-not-resuscitate (DNR) policy is well founded in end-of-life care. Of course, this is not yet a routine in India.

## PALLIATIVE CARE IS A LEGAL CHOICE

Legal aspects and human rights give the fundamental protections that allow equal participation and individual justice in a society.[4] It means *‘no one ought to harm another in his life, health, liberty or possessions’*. In the 20^th^ century, the right to healthcare is well-established, encompassing not only the delivery of basic clinical services but also an environment that allows good health to flourish. In this context, a terminal stage patient (or even family member) may often seek to end his/her life. Euthanasia is defined as ‘a deliberate intervention undertaken with the express intention of ending life to relieve intractable suffering’. The practice of euthanasia is legalized in some countries (The Netherlands, Belgium, some states of USA and Australia). However, euthanasia poses an ethical dilemma in palliative care. Simply said, ‘a doctor or nurse is not trained to deliberately end a patient's life’. It is interesting to note that the spread of palliative care, use of analgesics, and effective prescription of terminal sedation(even in the face of double effect) have reduced the need for euthanasia, in a recent Dutch study.[11] Hence, palliative care should be considered a better legal choice for the medical fraternity and the society.

## DILEMMA IN CARRYING OUT PALLIATIVE CARE RESEARCH

‘Two elderly patients, both with advanced lung cancer, have developed progressive dyspnea and chest pain. Can one be given oral morphine and the other fentanyl skin patch for two weeks to assess pain and symptom relief?’

Good medical practice requires evidence of effectiveness to address deficits in care. There are substantial opportunities to improve palliative care. However, a treating physician can face dilemmas, because research that involves patients near the end of life creates numerous ethical challenges.[12,13] Some of these dilemmas and challenges are real and some are perceived.

Inclusion of patients for palliative care research involves unique situations:

1. Dying patients are especially vulnerable
2. Adequate informed consent may be difficult to obtain
3. Balancing research and clinical roles is particularly difficult
4. Risks and benefits of palliative care research are difficult to assess.[13]

In practice, the dying of an incurable patient is medically recognized as a natural process. The patient can experience dynamic changes in physical and psychosocial symptoms. It is observed that there is prevalence of unrelieved symptoms such as pain, fatigue, dyspnea, constipation/bowel obstruction, depression/confusion and insomnia.[12] Much progress has been made in understanding and caring for most of these, yet greater research focus is still needed in many areas. Palliative care physicians and nurses should address existing deficits. Balancing the ethical principles in terms protecting the vulnerable patient from harm and at the same time carrying out scientifically designed studies should be possible.[12]

## CONCLUSION

Palliative care is mandated in advanced stage incurable cancer and other terminal chronic illnesses. The different aspects of palliative care such as pain and symptom control, psychosocial care, and end-of-life issues should be managed in an ethical manner. The cardinal ethical principles to be followed are-autonomy, beneficence, non-maleficence and justice. The palliative care experts and team members should carry out their responsibilities with honesty and dignity. Suffering due to unrelieved pain and unavailability of morphine are recognized as negligence of human rights. There are practical ethical challenges which need to be resolved. Truth telling, place of care, continuity of effective palliative care till the last days of life, confidentiality, use of antibiotics and blood transfusion, nutrition and advance directives can be the key points which confront a palliative care team. Progress in palliative care will come out of good research and medical professionals should undertake trials and studies in a legal and ethical manner. The delivery of palliative care and medical ethics are complementary, and use of the two together maximizes the protection and satisfaction available to the vulnerable patient and family members.

## References

[1] Beauchamp TL, Childress JF (2001). Principles of biomedical ethics.

[2] Joseph F (2006). A palliative ethic of care: Clinical wisdom at life's end.

[4] Peel M (2005). Human rights and medical ethics. J Soc Med.

[5] Thieren M, Mauron A (2007). Nuremberg code turn 60. Bull World Health Organ.

[6] Carlson RV, Boyd KM, Webb DJ (2004). The revision of the declaration of Helsinki: Past, present and future. Br J Clin Pharmacol.

[7] Beckman M (2007). 'Rock star' of biomedical ethics tackles cancer. J Natl Cancer Inst.

[8] (2004). NIH-State-of-the-Science Conference statement on improving end-of-life care. NIH Consens State Sci Statements.

[9] Myers J, Shetty N (2008). Going beyond efficacy: Strategies for cancer pain management. Curr Oncol.

[10] Fishman SM (2007). Recognizing pain management as a human right: A first step. Anesth Analg.

[11] van Der Heide A, Onwuteaka-Philipsen BD, Rurup ML, Buiting HM, van Delden JJ, Hanssen-de Wolf JE (2007). End-of-life practices in the Netherlands under the Euthanasia Act. N Engl J Med.

[12] Jubb AM (2002). Palliative care research: Trading ethics for an evidence base. J Med Ethics.

[13] Casarett DJ, Karlawish JH (2000). Are special ethical guidelines needed for palliative care research?. J Pain Symp Manage.

